# Health promotion for dementia risk reduction in Indigenous populations of Canada, Aotearoa New Zealand, United States of America, and Australia: Scoping review protocol

**DOI:** 10.1371/journal.pone.0309195

**Published:** 2024-08-26

**Authors:** Kathryn Meldrum, Yvonne Hornby-Turner, Valda Wallace, Sarah G. Russell, Rachel Quigley, Edward Strivens

**Affiliations:** 1 College of Medicine and Dentistry and Australian Institute of Health and Tropical Medicine, Cairns, Queensland, Australia; 2 Queensland Health, Cairns and Hinterland Hospital and Health Service, Cairns, Queensland, Australia; The University of Sydney, AUSTRALIA

## Abstract

Health promotion programs and strategies have the potential to support people to live healthier lives. Dementia, a collective name for brain disorders that impact thinking and memory, affects over 55 million people worldwide. Currently, there is no cure for dementia, so prevention is critical. Health promotion has the potential to reduce dementia by targeting the twelve potentially modifiable risk factors. A project currently being undertaken by the research team aims to strengthen the quality of clinical care and health services that specifically address dementia risk for Australian Aboriginal and Torres Strait Islander peoples. One of the intended strategies supporting the project’s aim is the need for appropriate and safe health promotion programs and resources that support dementia risk reduction. Consequently, the aim of this scoping review is to identify and determine the quality and appropriateness of existing health promotion programs and resources aimed at dementia risk reduction developed or modified for Indigenous populations of Canada, the USA, Aotearoa New Zealand, and Australia that could be incorporated into the broader project. The Joanna Briggs Institute method for scoping reviews will be used to identify programs and resources focussed on dementia risk reduction for Indigenous peoples. Searches will be limited to the English language and literature published since January 2010. Databases to be searched include: CINAHL, Medline, PsychInfo, PubMed, Scopus and Google. Data that answers the research questions will be extracted from the literature and recorded on a data charting form. A combination of quantitative and qualitative methods will be used to analyse the findings of the scoping review. Dissemination of the findings through continuing community engagement, conference presentations and publications will be led by Aboriginal and Torres Strait Islander members of the research team.

## Introduction

Health promotion enables people to better manage and consequently improve their health [1]. As both a social and political process, health promotion acts at an individual level by strengthening peoples’ skills and capability to lead healthier lives. It also acts to change the “social, environmental, and economic determinants of health” [1, p. 1580] to positively influence the health of the general public and as well as that of individuals. The five health promotion priority action areas proposed by the World Health Organisation’s (WHO) Ottawa Charter include policy, the environment, community action, personal skills and health service reorientation [1, 2]. Personal skills, that support people to understand how to change their behaviour, can be increased through health promotion interventions designed to focus on risk factors for disease [3]. However, a one-size-fits-all approach to health promotion may not be effective and cultural tailoring [4] may be necessary.

### Dementia

Dementia is an overarching name for a range of mostly progressive brain diseases that affect a person’s memory and other cognitive abilities as well as their behaviour to the extent that it impacts on their ability to sustain activities associated with daily living [5, 6]. In 2020, there were over 55 million people worldwide living with dementia [6]. Assuming no significant breakthrough in treatment, the number of people with dementia is projected to reach 139 million people by 2050 [7]. This is due to the increasing age of the global population as well as increasing risk factor prevalence [7]. Across the globe, dementia is a leading cause of disability and dependency amongst older people [5] and was the seventh highest cause of death in 2019 [7]. Dementia incurs significant financial impact globally [5]. It is estimated that up to 50% of these costs are hidden, borne by families and carers in their informal care of people living with dementia [7]. The WHO estimates that the global cost of dementia will increase to around 1.7 trillion US dollars in 2030 [8].

Increasing age and genetic factors are known risk factors for dementia. However, “dementia is not an inevitable consequence of ageing” [7, p. 117]. The Lancet Commission [9] found a growing body of evidence to support 12 potentially modifiable risk factors for dementia, which were incorporated into a life-course model of dementia. This was an increase from nine reported in 2017 [10]. The potentially modifiable risk factors for dementia were grouped into early life (<45 years of age), mid-life (45–65 years) and later life (>65 years). Less education is the early life risk. In mid-life, hypertension, traumatic brain injury, hearing loss, obesity, and consumption of more than 21 units of alcohol per week can contribute to increased dementia risk. Later-life contributors include smoking, depression, low levels of physical activity, diabetes, exposure to air pollution and social isolation [9]. In addition, other risk factors such as diet [11], renal function [12, 13], childhood trauma [14], and vision [15] are also being identified.

In summary, dementia currently affects a substantial number of people globally and is projected to increase as the number of older people in the population increases over time. Dementia also imposes a great financial burden on individuals, their communities and society in general. Currently there is no cure for dementia [7, 10]. Consequently, the WHO made dementia risk reduction the third of seven action areas of the global action plan [5] as prevention is critically important [16].

#### Dementia risk reduction

Reducing dementia risk is a priority for global public health [16] that can be addressed by appropriate health promotion interventions. It is estimated that up to 40% of cases of dementia may be reduced or delayed by addressing modifiable risk factors, particularly those arising in mid-life [9]. Additionally, non-communicable diseases (NCDs) such as depression, hypertension, diabetes, hearing impairment, high cholesterol and diabetes are also associated with dementia risk. Consequently, health promotion interventions targeting NCDs could also act to reduce dementia risk [7]. The WHO proposed that dementia risk reduction be grounded in scientific evidence and/or best practice [5]. Consequently, interventions should be: 1) person-centred; 2) cost-effective; 3) sustainable; and 4) affordable [17, p. 5]. They also need to be culturally appropriate [5].

### Context of the protocol and scoping review

The colonisation of Australia has historical and continuing impacts that are not limited to marginalisation, inter-generational trauma and racism that negatively influence the health and health-related outcomes of Australian Aboriginal and Torres Strait Islander Peoples [18, 19]. Notwithstanding these continuing impacts, the proportion of older Australian Aboriginal and Torres Strait Islanders is increasing, and projections indicate that this trend will continue [20]. Like the Indigenous peoples of Canada [21], the USA [22], and Aotearoa New Zealand [23], higher proportions of Australian Aboriginal and Torres Strait Islanders are diagnosed with age-related conditions including dementia, and at younger ages than the non-Indigenous population [24, 25]. A significant number of these age-related conditions are related to the ongoing impacts of colonisation, erosion of traditional lifestyles and social disadvantage [26]. Additionally. Indigenous peoples might be diagnosed with dementia earlier due to the cluster of risks, particularly NCDs, across the life course [7].

The context for this protocol and scoping review is a current project titled “Strengthening Primary Health Care Services to Prevent Dementia in Aboriginal and Torres Strait Islander Communities” [27] funded by the Australian National Health and Medical Research Council (APP 1172054). The aim of the broader project is to strengthen the quality of clinical care and health services that specifically address dementia risk. One of the intended strategies supporting the aim is the need for appropriate and safe health promotion resources for dementia risk reduction for Australian Aboriginal and Torres Strait Islander peoples.

### Rationale and aim

Flicker & Holdsworth [24] recommended that dementia be incorporated into pre-existing preventative health strategies and interventions. Primary health care centres in Australia proactively promote health, and prevent, diagnose, and treat disease and are therefore, ideally placed to deliver health promotion and dementia risk reduction strategies and programs.

The aim of the scoping review is to identify and determine the quality and appropriateness of existing peer-reviewed and grey literature on health promotion programs and resources aimed at dementia risk reduction developed or modified for Indigenous peoples of Canada, the USA, Aotearoa New Zealand, and Australia. The Indigenous peoples of these countries are the focus of this scoping review because they share a common history of being colonised by the British [28].

In line with the recommendations of Kjerland and colleagues [29], the authors define appropriateness as programs/interventions or resources designed or adapted with, by and for Indigenous peoples of the target population of the country. For example, the DAMPAA intervention [30] has been designed with and for Australian Aboriginal people and at least one of the team members identifies as an Australian Aboriginal person. However, it is important to highlight that the diversity of Indigenous peoples, known as First Nations, Metis or Inuit in Canada, Māori in Aotearoa New Zealand, American Indian, Alaskan Native and Native Hawaiian in the USA and Aboriginal and Torres Strait Islander People in Australia, across the globe [31] means that what is appropriate for one Indigenous group may not be appropriate for another. Additionally, the appropriateness of a program and/or resources can only be determined by a participant and may be expressed as how culturally safe they felt [32, 33] while engaging with the program and/or resources. The scoping review findings will identify appropriate programs and/or resources that incorporate the WHO principles for dementia risk reduction [5, 7] that could be incorporated into the broader project.

## Methods and analysis

The scoping review will be conducted according to the nine-step Joanna Briggs Institute (JBI) methodology for scoping reviews [34]. Both this protocol and the subsequent scoping review will be reported using the Preferred Reporting Items for Systematic Reviews and Meta-analyses Extension for Scoping Review (PRISMA-ScR) guidelines [35]. This protocol has also been written using the PRISMA-P checklist [36] (S1 File).

### Research question

The overarching research question guiding this scoping review is: What is the current evidence of programs/interventions and resources designed or modified for use with Indigenous populations living in Canada, Aotearoa New Zealand, USA or Australia that support dementia risk reduction?

Related sub-questions for this scoping review are:

1. How many programs/interventions and resources designed or adapted for Indigenous peoples are available?
2. What are the key characteristics of the programs/interventions or resources?
3. To what extent do the programs/interventions or resources adhere to the WHO principles?
4. What health promotion design approaches and strategies are being used?
5. What underlying theories, content, delivery, and timing approaches have been used to design the program/intervention or resource?
6. Have the programs/interventions or resources been designed with, for and by Indigenous peoples of the target population?
7. What is the quality of the programs/interventions or resources?
8. What is the evaluative evidence about the merit, value or worth of the programs/interventions or resources?

### Inclusion criteria

The Participant, Concept, Context (PCC) framework [34] has been used to guide inclusion criteria and search strategy for the proposed scoping review.

#### Participants

For the purposes of this scoping review participants of programs or interventions may be Indigenous populations living in Canada, Aotearoa New Zealand, USA or Australia. Although in some cases, programs/interventions or resources may have been designed for Indigenous peoples but their experience of using them may not have been reported.

#### Concept

The concept under consideration are programs/interventions or resources that support dementia risk reduction. As previously discussed, dementia risk reduction is one of the seven action areas proposed by the WHO Global action plan on the public health response to dementia 2017–2025 [5]. Health promotion approaches and strategies can be used to develop programs/interventions and resources that support dementia risk reduction. Specific data extracted from sources will include:

1. Key characteristics of the program/intervention or resource.
  - Name.
  - Indigenous population designed for.
  - Rationale for design.
  - How it was intended to be used.
2. Overarching health promotion design approaches and strategies used.
  - Objective(s) and target population.
  - Underpinning behavioural theory applied to design.
  - Type of intervention and strategy.
3. Specific dementia risk reduction design.
  - Underlying theory.
  - Content.
  - Delivery.
  - Timing.
4. Appropriateness of programs/interventions and/or resources for Indigenous peoples.
  - Designed or adapted with, for and by Indigenous peoples of the target population.
  - Inclusive of Indigenous peoples’ conceptualisation of health and wellbeing.
  - Utilises Indigenous peoples’ health promotion framework.
  - Utilises co-design approaches.
  - Participant’s perception of cultural safety.
  - Appropriate language and images.
5. Quality of the programs/interventions and/or resources
  - Reliability, validity, and appropriateness of program/intervention reporting.
6. Evaluative evidence of the programs/interventions and/or resources.
  - Merit, value or worth.
  - Effectiveness.
  - Efficiency.

Literature excluded from the data set will include secondary studies and those that do not include programs/interventions or resources that promote dementia risk reduction for Indigenous peoples living in Canada, Aotearoa New Zealand, USA or Australia. The rationale for excluding secondary studies is that although they may report the design of and/or outcomes of a program/intervention or resources, findings have not been collected by the authors. Consequently, they cannot provide a rationale for or any commentary on the development or design of the program/intervention or resource.

### Context

The context of the scoping review is Indigenous populations living in Canada, Aotearoa New Zealand, the USA, or Australia.

### Types of sources

This scoping review will consider both experimental and quasi-experimental study designs using quantitative, qualitative, or mixed methods. Additionally, grey literature that reports or contains (outlines) a program/intervention or resource will be included. For example, The Alzheimer Society of Canada – Dementia Information for Indigenous peoples; First Nations, Métis and Inuit. Critical reviews, literature reviews and systematic reviews will not be included in the data set. However, their reference lists will be reviewed for relevant primary data sources.

The following inclusion criteria will guide identification of possible sources:

1. Sources focussed on a program/intervention or resource designed or adapted to promote dementia risk reduction in Indigenous peoples living in Canada, Aotearoa New Zealand, the USA, or Australia.
2. Identifies the design approach used to develop the program/intervention or resource.
3. Uses primary data sources.
4. Program/intervention or resource designed for use in any setting (primary health care, community).
5. Constitutes a complete paper, report, or evaluation (not an abstract, executive summary etc.)

The scoping review is registered with the Open Science Framework (https://osf.io/kzax4/)

### Search strategy

Published and unpublished studies, reports, evaluations, and resources will be located through the search strategy. This review will use a three-step search strategy [34]. In the first step an initial limited search of MEDLINE (Ovid) and CINAHL identified articles on the topic. Words and index terms of relevant articles supported the development of a full search strategy. A pilot search strategy is provided in S2 File which includes the search terms for the grey literature. This step was supported by Subject-specific liaison librarians. In addition, reference lists of included sources will be reviewed.

Published or unpublished studies and other data sources that have been written in the English language will be included in the data set. Only sources published since January 2010 to present will be included. Only English language sources will be included in the data set because it is the first language of authors conducting the searches and screening the data set. Literature since 2010 will be used because, prior to that year there was limited information about the range of dementia risk factors. Additionally, the WHO only made health promotion one of the dementia risk reduction action areas since 2017, so there is likely to be less relevant programs/interventions or resources available prior to that year.

Sources will be located by searching: CINAHL; Informit (Health and Indigenous Peoples Collection); Medline (Ovid); PsychInfo; PubMed (Ovid); and SCOPUS databases. Search strings will be archived on searchRxiv (https://searchrxiv.org/) to facilitate efficient reporting in the scoping review. Sources of unpublished studies and grey literature will be located using Google, Google Scholar, and website searches of key dementia organisations in each of the included countries. Google and Google Scholar searches will use incognito/private browser mode with cookies and cache cleared prior to the search. Only the first five pages of results for each search will be reviewed for inclusion in the data set.

### Study/Source of evidence selection

In step two all potential source citations will be uploaded into EndNote (Version 20.1) using respective database citation manager/export function. The method proposed by Peters [37] will then be used to manage potential sources. Subsequently pilot test will be conducted [34]. Following pilot testing, two independent reviewers will screen titles and abstracts against the scoping review’s inclusion criteria. The same two independent reviewers will then retrieve full-text versions of potentially relevant sources and assess them for inclusion in the data set. An adapted Preferred Reporting Items for Systematic Reviews and Meta-analyses extension for scoping review (PRISMA-ScR) flow diagram [38] will be used to detail reasons that any source was excluded. The PRISMA-ScR flow diagram will be included in the subsequent scoping review.

Disagreements between reviewers that arise at any stage of the source selection process will be resolved through discussion. An additional reviewer/s will be asked to review the source if agreement cannot be achieved.

### Data extraction

Specific information about the participants, concept, context, and key findings relevant to the review’s objective and questions will be extracted from sources and recorded in an Excel database developed by the first named author. The database will be iteratively reviewed by both reviewers during data extraction and additional fields will be added if necessary. Additionally, a quality appraisal of relevant data sources will be conducted using the Public Health Ontario Meta-tool for Quality Appraisal for Public Health Evidence (Meta QAT) [39, 40]. Quality appraisal is not usually conducted in scoping reviews [34]. However, the rationale for this scoping review is to identify dementia risk reduction programs/interventions or resources that could be incorporated into a project currently underway with Australian Aboriginal and Torres Strait Islander Peoples. Therefore, it is important to identify which programs/interventions or resources are of good quality and appropriate for the population. Good quality and appropriate programs/interventions or resources will then be pooled for potential incorporation into the current project.

Disagreements will be managed as previously described. Authors of sources will be contacted to request missing or additional data or other information regarding resources and local context of such resources, if necessary.

### Data analysis and presentation

A combination of quantitative and qualitative methods will be used to analyse the findings of the scoping review. Quantitative summing will be used to identify the number of programs/interventions and resources. Qualitative coding to develop themes [41] will be focussed on:

- Key characteristics of each program/intervention or resource.
- Overarching health promotion design approaches and strategies.
- Specific dementia risk reduction design approach.
- Appropriateness of programs/interventions and/or resources for Indigenous peoples.
- Quality of the programs/interventions and/or resources.
- Evaluative evidence of the programs/interventions and/or resources.

Graphic, diagrammatic, or tabular approaches will be used to present findings in the scoping review. Tabulated and/or charted findings will be supported by a narrative summary. The narrative summary will also discuss how the findings relate to the scoping review’s objective and answer the research question and sub-questions.

### Ethical considerations

This scoping review is being conducted as part of a broader project, which has ethical approval from Queensland Metro South Health Service District Human Research Ethics Committee (HREC)(PR/2023/QMS/61828) and James Cook University HREC (H8599).

### Personal and public involvement and engagement

The broader project was developed following feedback from community and health service staff during the team’s work on the Queensland sites for the Community Health Approaches To) Dementia in Aboriginal and Torres Strait Islander Communities (“Let’s Chat”) project. In Let’s Chat, the team worked with Aboriginal Health Services to improve detection of cognitive impairment and dementia, as well as dementia care and brain health [42]. Feedback was that strategies were needed to address the increased risk of dementia seen in Aboriginal Communities that could be embedded into primary health care.

## Discussion

This study will support the identification of and determine the quality and appropriateness of dementia risk reduction programs and/or resources designed or adapted for Indigenous peoples of Canada, Aotearoa New Zealand, USA, and Australia. The findings of the scoping review will identify programs and/or resources that could be integrated into a current project.

A scoping review is appropriate for this study because it aims to identify all dementia risk reduction programs and/or resources designed or adapted for Indigenous peoples that are published or available in the grey literature. While determining the quality of included literature is not normally undertaken in a scoping review, we intend to use the Meta—QAT [39, 40] to review and report on program and resource quality. In addition, ensuring that programs and resources are appropriate for Australian Aboriginal and Torres Strait Islander Peoples will also be undertaken in the scoping review.

### Limitations

There are two identified limitations of this scoping review. First, data collection will be limited to literature published in the English language. This will mean that literature published in a language other than English will not be included in the data set. Second, data collection will be limited to literature published since January 2010. We anticipate that this limitation will not be a significant issue as information about the 12 potentially modifiable risk factors for dementia across the life-course is relatively recent. Therefore, programs and/or resources that have been developed more recently are more likely to reflect the latest scientific evidence and good practice principles that the WHO recommends [5].

### Dissemination plan

Dissemination of the scoping review findings will be led by Aboriginal and/or Torres Strait members of the research team. A range of strategies including ongoing community engagement, conference presentations and publications will be used to support dissemination. Knowledge translation will include incorporating the scoping review’s findings into the “Strengthening Primary Health Care Services to Prevent Dementia in Aboriginal and Torres Strait Islander Communities” [27] project.

## Supporting information

S1 File PRISMA-P checklist. (PDF)

S2 File Pilot search strategy. (PDF)
