# Supplementary material for: Health promotion for dementia risk reduction in Indigenous populations of Canada, Aotearoa New Zealand, United States of America, and Australia: Scoping review protocol
Source: PLoS One. 2024 Aug 26;19(8):e0309195. doi: 10.1371/journal.pone.0309195 (PMC11346915; doi:10.1371/journal.pone.0309195)
Supplement: S2 File — (PDF) [file pone.0309195.s002.pdf]

S2 File

Pilot search strategy

Search terms used in PubMed database search

dementia AND Indigenous peoples OR Australian Aboriginal and Torres Strait Islander peoples OR American Indian or Alaska Native OR Maori people OR Native Hawaiian OR First Nations People* OR Metis AND dementia risk reduction

12 records returned

Search terms used in CINAHL database search

Dementia AND (MH "Indigenous Peoples+") AND “preventative health services” OR “risk reduction”

0 records returned

Grey literature search strategy

Google (incognito window) (48 citations recorded)

dementia AND Indigenous peoples OR (Australian Aboriginal and Torres Strait Islander peoples) OR (American Indian or Alaska Native) OR Maori OR Native Hawaiian OR (Canadian First Nations) OR Metis OR Inuit AND dementia risk reduction Date limits 2010-2024
